# Organic Honey from the Middle Atlas of Morocco: Physicochemical Parameters, Antioxidant Properties, Pollen Spectra, and Sugar Profiles

**DOI:** 10.3390/foods11213362

**Published:** 2022-10-26

**Authors:** Toufik Bouddine, Hassan Laaroussi, Meryem Bakour, Ibtissame Guirrou, Farid Khallouki, Hamid Mazouz, Hassan Hajjaj, Lhoussain Hajji

**Affiliations:** 1 Bioactive and Environmental Health Laboratory, Moulay Ismail University of Meknes, Meknes 50050, Morocco; 2 Cluster of Competency of Agri-Food and Food Safety, Moulay Ismail University of Meknes, Meknes 50050, Morocco; 3 Department of Biology, Faculty of Sciences Dhar El Mahraz, University Sidi Mohamed Ben Abdallah of Fez, Fez 30000, Morocco; 4 Regional Agricultural Research Center of Meknes, National Institute of Agricultural Research, Meknes 50050, Morocco

**Keywords:** honey quality, Middle Atlas of Morocco, physicochemical characterization, sugar contents, melissopalynological analysis, antioxidant potential

## Abstract

This work aimed to characterize and compare the physicochemical, ascorbic acid, phenolic, and flavonoid compounds, as well as the antioxidant properties, pollen spectra, and sugar profiles of twenty-three organic honeys produced in the Middle Atlas of Morocco. As results, the pollen analysis showed 22 taxa and revealed the dominance of *Ziziphus lotus* pollens for all monofloral honeys. The moisture content ranged from 15.9 to 19.0%, pH values werebetween 3.9 and 4.8, electrical conductivity varied from 100 to 581 µs/cm, ash content varied from 0.1 to 2.4%, and the invertase activity ranged from 3.5 to 36 U/kg. Moreover, hydroxymethylfurfural(HMF) varied from 1.2 to 13.5 mg/kg, which confirmed the freshness of our honey samples. For the sugar profiles, there were no significant differences between the examined groups of honeys (*p* > 0.05) for both fructose and glucose. Additionally, our study showed good antioxidant properties (total antioxidant activity ranged from 34.18 to 131.20 mg AAE/g; DPPH IC_50_ values ranged from 8.14 to 45.20 mg/mL; ABTS IC_50_ values ranged from 8.19 to 32.76 mg/mL) and high amounts of phenolic compounds ranging between 20.92 ± 0.03 and 155.89 ± 0.03 mg GAE/100 g, respectively; flavonoid compounds ranged from 5.52 to 20.69 mg QE/100 g, and ascorbic acid ranged from 8.01 to 23.26 mg/100 g. Overall, the proximate composition and the general characterization of organic monofloral and polyfloral honeys as sustainable and health-promising functional products may increase their commercial values, promote their marketability, and might have a significant impact on the basic circular/sustainable economy as a solid lever for solidarity economic development, especially in the rural/poor Moroccan communities. The investigated features may allow and support the incorporation of Moroccan organic honeys and their biovaluable ingredients in the nutraceutical and food industries for multiple purposes.

## 1. Introduction

Honey is a fluid, pasty, or crystalized functional food collected by bees from blossom nectar or sweet deposits (honeydew) from living plants, which isthen modified and stored in honeycombs. Honey is used in the treatment of several debilitating diseases [1,2], mainly due to the attributes of its enzymes and polyphenolic compounds.

The beekeeping sector is one of the most important economic activities in Morocco, elaborating several products such as honey, pollen, propolis, beeswax, and royal jelly. Indeed, honey production in Morocco exceeded 7000 tons in 2018 [3]. Due to its important floristic, faunal, and landscape diversity, Morocco is endowed with an important and unique beekeeping potential, giving it great originality which makes it one of the most interesting regions in terms of biological and biogeographic richness [4]. Likewise, as a Mediterranean country, Morocco is known for its healthy diet and traditional pharmacopeia, in which bee products, especially honey, a sacred food product, has been used as a biofunctional dietary sugar to prevent and treat several human health disorders [5,6]. To date, the importance given to honey has contributed to the modernization of the sector through the leverage effect of the Green Morocco Plan (GMP) as well as the National Initiative for Human Development (NIHD). Furthermore, in its strategy to promote local products, the Moroccan Ministry of Agriculture has set a referential catalog for high-quality terroir products [7].

The Moroccan Middle Atlas has great potential for the production of high-quality organic honeys, which are rich in several bioactive metabolites due to the diversity of medicinal and aromatic plants present in such a region. In our region of study, it should be noted that the honeys labeled organic are currently made up solely of rosemary (50%), *Ziziphus lotus* (25%), and *Bupleurum* (25%) from the total annual production [8]. The area is well-recognized for its high plant diversity and high levels of endemism, exhibiting it as a priority site for conservation in the Mediterranean region [9]. Honey contains complex and diverse molecules with varying proportions, some of which have strong antioxidant properties [10]. Moreover, great interest has recently been brought to their characterization and contribution to organoleptic qualities [11]. The inspection for new characteristics of quality models in natural honeys is currently experiencing unprecedented enthusiasm for its major interest in the labeling of food products. For this reason, there is a myriad of global research studies on the characteristics of honey [12,13,14].

Most research works on Moroccan honeys have focused on physicochemical characterizations [5,15,16]. However, there is a lack of combined scientific characterization data with product quality indicators. Therefore, it remains crucial to carry out a more complete profile of the product by introducing its main organoleptic patterns appreciated by consumers. These grounds include, but are not limited to, aroma, color, texture, and the botanical and geographical origin that give the product its added nutritional value. Pollen grains in honey are indeed good indicators of the flowers that bees have encountered on their pollination and nectar-gathering journeys. Thus, the study of pollen profiles makes it possible to directly trace the plant species’ source and predict its geographical origin. The promotion of organic honeys from the Middle Atlas is, in itself, a way of promoting aromatic and medicinal plants, thus underlining the importance of the floristic biodiversity of a major production region in Morocco. This work aimed to define some physicochemical and palynological characteristics, sugar contents, and antioxidant potentials of local organic honey from the Moroccan Middle Atlas region, according to their geographical areas of collection and floral richness. A sensory evaluation as a support for its first authentication to help detect any potential honey adulteration was conducted.

## 2. Materials and Methods

### 2.1. Chemical Reagents

The following compounds were purchased from Sigma-Aldrich: D-glucose ≥ 99.5%, D (−) fructose ≥ 99%, D (+) Turanos ≥ 98%, erlose ≥ 94%, isomaltotriose, D (+) melibiose ≥ 99,0%, D (+), raffinose pentahydrated ≥ 98.0%, palatinose hydrate ≥ 99%, sucrose ≥ 99.5%, D (+) maltose monohydrate min 98%, melezitose ≥ 99.0%, trehalose dihydrate (Certified Reference Materials), D-panose ≥ 88 97%, maltotriose, isomaltose 98%,ascorbic acid, gallic acid, 2,2-diphenyl-1 picrylhydrazyl (DPPH), Folin–Ciocalteu’s reagent, hexamethyldisilazane ≥ 99%, trifluoroacetic acid, hydroxylamine hydrochloride, acetic anhydride, pyridine, potassium hexacyanoferrate (II) trihydrate, and zinc acetate dihydrate. Methanol, sulfuric acid, ethanol, and acetonitrile were purchased from Merck (Darmstadt, Germany). Aluminum chloride, sodium carbonate, sodium hydroxide, and sodium nitrite were purchased from Merck (Darmstadt, Germany). All chemicals used were of analytical grade.

### 2.2. Honey Samples

Twenty-three (*n* = 23) honey samples (500 g) were collected between the first of April and the end of September 2018 from modern and healthy hives installed in different ecogeographical regions of Morocco (15 areas) located in the north, center, and south of the Middle Atlas (Morocco). All samples were centrifuged upon collection and stored at 4 °C until analysis.

Four samples were harvested from three localities in the north of the Middle Atlas (Ain cheggag, Ahl Sidi Lahcen, and Ifrane); 16 samples were harvested from 10 regions in the middle of the Middle Atlas (Serghina, Boulemane, El Mers, ImouzarMarmoucha, Ouled Ali Youssef (Imouzar), Guigou, Bouiblane, Enjil, Skoura, and Baqrit-Timhdit); 3 samples were harvested from two sites in the south of the Middle Atlas (Outat el haj and Missour) (Table 1).

### 2.3. Melissopalynological Analysis

The melissopalynological analysis was conductedqualitatively using the acetolysis method of subsamples with acetic anhydride and sulfuric acid (1.8 mL/0.2 mL) at 70 °C until the pollen grains turned brown [17]. Each honey sample (10 g each) was diluted in 100 mL of distilled water, and clarified by centrifugation at 2500 rotations per minute for 5 min. The supernatant was discarded, and the pellet was suspended in 10 mL of glacial acetic acid and centrifuged again. For each sample, at least 400 pollen grains per sample were counted and identified according to the standard general key of pollen types, and then compared with the pollen-source catalogs of flowers in the study area [18,19].

Moreover, with the aid of pollen atlases, different bibliography sources were consulted [20,21,22,23,24]. The results of pollen frequencies from various representative plants or families in each sample were collected and recorded. Frequency ranks were determined by dividing the percentage of pollen grains into dominant pollen species (>40%), minor pollen species (16–40-10%), important minor pollen species (10%), and minor pollen types 3%) [25].

### 2.4. Routine Analysis and Proximate Composition

Analyses of physicochemical properties were carried out on the 23 honey samples. They were analyzed usingthe harmonized methods of the international honey commission (IHC) proposed by Bogdanov et al. [26]. These properties include moisture, electrical conductivity, ash content, pH, free lactonic acidity, and invertase activity.

Water activity was measured with a digital HygroPalm 23-AW water activity meter (Rotronic, Bassersdorf, Switzerland). For each determination, two replicates were obtained and the average value was used.

Honey color was analyzed after being heated to 50 °C to dissolve sugar crystals, and the color was determined by spectrophotometric measurement of the absorbance of a 50% honey solution (*w*/*v*) at 635 nm. The honey samples were classified according to the Pfund scale after conversion of the absorbance values in mm.

Pfund = −38.70 + 371.39 × Abs(1)

where mm Pfund is the intensity of honey color in the Pfund scale and Abs is the absorption of the honey solution. The colorimeter is compared with Pfund’s scale.

### 2.5. Hydroxymethylfurfural (HMF) Determination

The HMF content was determined by HPLC Agilent 1200 system with LC-20AT quaternary pump, Degasser DGU-20A5, SIL-20AC autosampler, SPD-M20A diode array detector, CTO-20AC Column Oven, software Shimadzu Client/Server, Version 7.3 from Shimadzu Corporation (Tokyo, Japan), as was described by da Silva et al. [27]. Briefly, in a flask (50 mL), 3 g of honey was weighed and dissolved in 25 mL of Milli-Q water. To clean up the solution, 0.5 mL of Carrez I (potassium hexacyanoferrate) solution, and then 0.5 mL Carrez II (zinc acetate dehydrate) were added and stirred. Next, the flask was immediately filled up to a mark with Milli-Q water and the solution obtained a milky color.Depending on the type of honey, the color varied from light yellow to brown. The contents of the flask were filtered through a 5 µm filter paper and the first 10 mL of the filtrate was discarded. Then filtrate was refiltered over a 0.45 μm polytetrafluoroethylene (PTFE) filter and transferred to the glass vials before chromatography.

Chromatographic conditions: The column used was a ZORBAX Eclipse XDB-C18, reverse phase (Supelco, Bellefonte, PA) stainless-steel column (150 mm × 4.6 mm; film thickness 5 μm);injection volume: 25μL; the flow rate of the mobile phase: 0.7 mL/min—isocratic; mobile phase: 90% (1% formic acid in water); 10% acetonitrile; column temperature: 30 °C; DAD detector λ = 285 nm; run time: 15 min. Serial standard solutions of HMF (1–50 mg/L) were made in Milli-Q water.

### 2.6. Total Phenolic Content (TPC)

The method of Folin–Ciocalteu described by Singleton [28] was used for the polyphenols quantification. Gallic acid (0–500 mg/L) was used as a standard to achieve the calibration curve (R^2^ = 0.996). The results were expressed in mg gallic acid equivalent (GAE) per 100 g of honey (mg GAE/100 g).

### 2.7. Total Flavonoid Content (TFC)

Total flavonoid content was determined following the method described by Sousa et al. [29]. A range of quercetin standards (0.5–100 mg/L) were prepared to produce the calibration curve, which was analyzed in the same manner as that for the honey samples. Then, 2 mL of 2% AlCl_3_ reagent was added to 2 mL of honey solution (1 g/10 mL). After 30 min of incubation period at room temperature, the absorbance was determined spectrophotometrically at 420 nm. The TFC was calculated for each honey sample using the regression equation (y = 0.0247x + 0.0173) from the standard curve generated by quercetin (R^2^ = 0.999). The calculated values were recorded as mg of quercetin equivalent (QE) per 100 g of honey.

### 2.8. Ascorbic Acid Quantification

To quantify the ascorbic acid (vitamin C) contents in honey samples, the method described by Nweze et al. was used with slight modifications. Briefly, 5 g of honey was mixed with 10 mL of distilled water and the following solution was prepared: 1% starch indicator solution, vitamin C standard solution (0.001 g/mL of distilled water), and 0.268 g potassium iodate (KIO_3_) and iodine solution (prepared in a 500 mL beaker by mixing 5 g potassium iodide (KI) with 200 mL of distilled water; 30 mL of 3 molars of sulfuric acid was added into the beaker and diluted with distilled water until it produced 500 mL of solution). Standardization of iodine solution with the vitamin C standard solution was by mixing 25 mL of vitamin C solution with 10 drops of 1% starch solution and then titrating against iodine solution until a blue-black color was observed. Titrations were repeated in triplicates. The volume of the honey sample used in the titrations was measured and the concentration of ascorbic acid in mg per 100 g of honey was calculated [30].

### 2.9. Total Antioxidant Capacity

The total antioxidant capacity of honey samples was evaluated by the phosphomolybdenum method described by El-Haskoury et al. [5]. A total of 5 g of honey was mixed with 10 mL of distilled water. From this solution, 25 µL was mixed with 1 mL of reagent solution (0.6 M sulfuric acid, 28 mM sodium phosphate, and 4 mM ammonium molybdate). After 90 min of incubation in a water bath at 95 °C, the absorbance of the solution was measured at 695 nm against blank. Ascorbic acid was used as the standard calibration. The results were expressed as milligrams of ascorbic acid equivalent per gram of sample.

### 2.10. Free-Radical-Scavenging Activity (DDPH Assay)

The DPPH radical-scavenging activity was determined following the method described by Ferreira et al. [10]. Briefly, 300 µL of each honey sample of different concentrations (3.90 to 125 mg/mL) was added to 2.7 mL of 150 µM DPPH-methanol solution with an absorbance of 0.700 ± 0.01 at 515 nm, and the absorbance of the mixture reactions was measured at 517 nm against a blank after 1 h of incubation period at room temperature.The antiradical activity (% inhibition) was calculated using the equationbelow. Honey sample concentration providing 50% inhibition (IC_50_) was calculated according to the linear regression algorithm of the plotted inhibition graph percentage.

(2) $$\%\;\mathrm{inhibition}=\left[\left(\mathrm{Abs}\;\mathrm{control}\;-\mathrm{Abs}\;\mathrm{sample}\;\right)/\mathrm{Abs}\;\mathrm{control}\;\right]\times 100$$

Ultra-pure water was used as a control solution instead of the honey, and a standard solution of 6-hydroxy-2,5,7,8-tetramethylchroman-2-carboxylic acid (Trolox) was used as a positive control with a concentration range of 250–15 µM, R^2^ = 0.997.

### 2.11. Radical Cation Decolorization (ABTS Assay)

The ABTS assay of different honey samples was determined as follows: 2 mL of 2,2′ -azino-bis (3-ethylbenzothiazoline-6-sulfonic acid) diammonium salt (ABTS)-radical-cation solution was mixed with 100 μL of different dilutions of each honey solution (0.97 to 125 mg/mL). The resulting solutions were incubated in the dark for 30 min at room temperature and the intensity of produced coloration was measured immediately at 734 nm by UV-Vis spectrophotometer (Jasco V-730) [31].

Trolox (800–30 µM, R^2^ = 0.998) was used as a positive control. The ABTS-radical-cation-inhibition percent was determined using the equation of DPPH.

### 2.12. GC-FID Determination of Sugars

Sugars were determined by gas chromatography with flame ionization detection (GC-FID), according to Pierce–Portallier’s method [26]. The sugars analysis was carried out using two-step derivatization procedures (oximation and trimethylsilylation).

In a flask of 500 mL, we added 5 mL of mannitol and 3 g of each honey sample dissolved in distilled water. Each flask was then filled to the mark with distilled water. After thorough mixing of samples for 10 min, 100 μL of each preparation was transferred to a conical-bottomed test tube. It was allowed to dry at 50 °C under a stream of nitrogen. After that, 200 μL of oximation solution (0.06 g of hydroxylamine chloride dissolved in 5 mL of pyridine) was added and test tubes were well-sealed with a screw-on plug. Mixtures were homogenized and heated at 65°C and mixed at 1400 rpm for 30 min. The oximes obtained in this step were silylated with hexamethyldisilazane (100 µL) and trifluoroacetic acid (10 µL) at 25°C for 30 min. After centrifugation, the trimethylsilyl derivatives were separated and quantified by gas chromatography. AUTOSYSTEM XL FID, PERKIN ELMER Auto system XL with detector FID (Perkin-Elmer, Shelton, CT, USA). A 0.6μL injection of each sample was analyzed under the following GC conditions: initial oven temperature of 70 °C, then programmed at 49 °C/min from 70 to 140 °C and from 140 to 300 °C at 6 °C/min with helium as carrier gas [32]. Data acquisition and analysis of the chromatographic peak areas were carried out using turbochrom navigator software. For qualitative analysis, retention times relative to mannitol both for standards and sample peaks were used [26]. Honey samples were also spiked with standards in order to first verify the identity of the chromatographic peaks and then to quantitate every identified sugar.

Duplicate injections were performed, and average peak areas were used for the peak quantification. Glucose, fructose, sucrose, maltose+, turanose+, melibiose, isomaltose, trehalose, palatinose, raffinose, erlose, melezitose, maltotriose, and panose were used as standards. The acquisition was completedusing turbochrom navigator software operating in a windows environment.

### 2.13. Statistical Analysis

The data are presented as means ± SD. Correlations between the parameters studied were achieved by the Pearson correlation coefficient (r). The statistical calculations (analysis of variance and PCA) and the least-significant difference (LSD) according to Student–Newman–Keuls were used to compare and separate the means, and significance was accepted at the 5% level. Comparisons of treatment means (LSD, 5% level) were conductedusing the SPSS 22 statistics software (IBM Software Lab, Rome, Italy).

## 3. Results and Discussions

### 3.1. Melissopalynological Analysis

Twenty-two (*n* = 22) pollen taxa were identified in the 23 honey samples and represented two classes of pollen frequency. Nine pollen taxa werevery predominant and eighteenweresecondarily dominant. The analysis of the pollen spectrum highlighted 16 honey samples, distributed into 9 groups, with pollen frequencies of more than 40% (Table 2). *Ziziphus lotus* pollens (G1) dominated in six honey samples, Rhamnaceae (G2) and *Sinapis arvensis* (G3) in two honey sampleseach, and the pollen spectraof Fabaceae (G4), *Ammi visnaga* (G5), Apiaceae (G6), Lamiaceae (G7), *Rosmarinus officinalis* (G8), and *Thymus vulgaris* (G9) were predominant in one honey each (Table 2). Other samples (seven) were all-flower honeys (G10) without apparent pollen dominance, made up of secondary pollens of one to four taxon maximum, with pollen frequencies between 10 and 36%. The total of 23 honey samples had a great pollen diversity, which reflects the floral diversity of the Middle Atlas and flavors the production of honey with different characteristics. The honey samples collected in the study area, representing a large analytical area, generally contained monofloral honey. Sixteen honey samples were identified as monofloral with a quantity superior to 40% and seven other honeys wereregrouped as multifloral. All honey samples in this study showed that the Middle Moroccan Atlas area contains very important aromatic and medicinal species for the production of nectar, with a fairly long flowering period from April to September. As for the origins, the results for the honey types are in line with the beekeepers’ proclamations.

### 3.2. Physicochemical Analysis

The moisture content of honey is likely to be associated with the harvest time and the level of its maturity in the hive. This parameter is highly important for the shelf-life of the honey during storage. The Codex Alimentarius honey standards [33] set the maximum value of this parameter at 20%. In our study, the differences between the analyzed samples may be due to several factors and, to name only a few, these include environmental conditions, harvest period, degree of maturity reached in the hive, moisture of the nectar of the original plant, and the processing of the honey during the extraction process and storage [20]. The moisture content of all analyzed honey samples was below the maximum limit (20%) fixed by the Codex Alimentarius, in which values ranged from 14.2 to 19.4%, indicating the maturity of all harvested samples. The obtained values are in agreement with those reported by ElSohaimy and coworkers, who analyzed honey collected from different middle eastern areas, in which Saudi Arabian (*n* = 65), Egyptian (*n* = 66), Yemeni (*n* = 63), Emirati (*n* = 72), and Iraqi (*n* = 53) honeys showed water content values of 14.6, 14.96, 16.1, 16.26, and 17.06%, respectively [34].

pH is another important parameter affected by apicultural practices and storage conditions. This parameter influences the stability and shelf-life of honey [16]. As shown in Table 3, all examined honeys revealed an acidic character. The pH values ranged between 3.89 and 4.37, except for the simple H15 (6.34), which exceeded the limit pronounced by the Codex Alimentarius. These values were similar to those reported previously for the Tunisian honey samples (values ranged from 3.67 to 4.11) [35], along with Algerian, Spanish, and Portugal honeys that have been found to vary between 3.50 and 4.58 [36,37,38]. In general, the international norms corroborate our documented values [39].

In line with this parameter, the free acidity values of all honeys were between 12.34 and 49.02 mEq/kg.These results are below the limits reported by the Codex Alimentarius (<50 mEq/kg) [33] and are in line with those obtained by Silva et al. for Portuguese honeys [40]. Acidity is an important criterion of quality, which strongly depends on honey age and its specific composition of aliphatic and aromatic acids. Total acidity values in the analyzed honeys ranged from 22.85 to 77.94 mEq/kg. This indicates the absence of undesirable fermentation. Our obtained values are also higher than those reported by Achour et al., varying between 10 and 40 mEq/kg [41].

The content of lactone in honey samples was between 7.07 and 28.92 mEq/kg. These results are more dispersed than those obtained by Elamine et al. [42] (between 14.00 and 18.50 mEq/kg).

Electrical conductivity (EC) represents the ability of a body to allow the passage of electrical currents. It depends on the mineral content and acidity of the honey [26]. This parameter shows a great variation between the examined samples according to the honey’s floral origin [16]. The EC ranged from 100 to 820 µs/cm, with the lowest value referred to the monofloral honey sample (*R. officinalis*, 64%) collected from the ImouzerMermoucha area, with an EC = 100 µs/cm. However, the highest value corresponded to the asfour honey sample (H7) from Ain cheggag station, with an EC = 840 µs/cm. Hence, the results below show a wide variation depending on the floral origin. These values are below the maximum value of 800 µs/cm for honey from floral (blossom) origin, as specified in the Codex Alimentarius and E.U. Council [33,43]; honeys with an EC >800 µs/cm are honeydew honeys (except for chestnut honey). These results conform with previous investigations, in which electrical conductivity varied from 215 to 780 for eight samples from different localities in Morocco [44], as well as from 270 to 850 for twenty samples of honey from Morocco in other studies [16,45]. Similar results have been documented by Bayram and coworkers for honey samples collected from different ecogeographical areas of Turkey, in which EC varied between 286 and 800 µs/cm [14].

In terms of mineral content (ash), this parameter varies according to the different botanical geographical origins. Our results indicate that most analyzed honeys showed values less than the acceptable limit set by the Codex Alimentarius (<0.6%) [33], except the samples H3, H8, and H9, respectively, from Ain cheggag, Boulemane, and Imouzzer. In other recent studies, the analysis of honeys collected from the same areas of our harvests (Middle Atlas, Morocco), showed ash contents of 0.21–0.55% and 0.13–0.32%, respectively, for the analyzed honeys by Laaroussi et al. [46] and El Amine et al. [47].

Hydroxymethyl-furfural (HMF)content is broadly recognized as a parameter affecting honey freshness and purity [33]. This criterion should not exceed 40 mg/kg [43]. The value of our samples wasless than 35.70 mg/kg. All samples had an HMF value below the recommended limit of 40 mg/kg. In the same line, Smetanska and coworkers investigated the physicochemical quality of organic honeys from different middle eastern areas and showed values below 40 mg/kg [48]. Accordingly, these samples had HMF contents of 10.94 mg/kg for Egyptian honey with a predominance of *Trifoliumalexandrinum* L. pollen, 12.82 mg/kg for Yamani (*ZiziphusSpina-christi* L.), and 22.47 mg/kg for Saudi Arabia (*Ziziphus spina-christi* L.). Generally, several factors influence the formation of HMF, such as storage conditions [16,49,50]. Moreover, it is well known that heating contributes to the formation of HMF, which is produced during the acid-catalyzed dehydration of hexoses, especially coming from fructose and glucose [49,51].

The color of honey is an important indicator reflecting its richness in multitudes of components, such as polyphenols and carotenoids, among others. In our honey samples, the observed colors varied from water white (8.1 mm Pfund) in the honey H13 (Figel) from Guigo to dark amber (125.7 mm Pfund) in the honey H18 (Jujube) from Missour, explaining the variability seen amongst the analyzed samples. These results are in agreement with those reported by El amine et al. [42].

Surrounding the TSS values, such a parameter—which is inversely proportional to the moisture—varies between 80.6 and 85.8%. The sample from Serghina, Boulemane, center of both thyme and multifloral at once, presents the highest value of dry matter (84.2%), while the sample of the lharra type from Oumjniba presents the lowest value (80.6%).

In our study, water activity values varied from 0.507 to 0.596. This quality parameter is mainly dependent on the glucose content [44], which is related to some factors including the floral source of the nectar, geographical origin, climatic conditions, season of the year, processing and storage conditions, and the degree of maturity of the honey reached in the hive, among many [52].

Honey is a supersaturated sugar solution with a low water activity (aw), which means that there is insufficient water available to support the growth of bacteria and yeasts (although some osmophilic yeasts can survive in very low water content, causing spoilage of the honey). This factor is important for determining the enzymatic activity, survival, and the limitation of the growth of microorganisms and the product deterioration by fermentation. Furthermore, this parameter is influenced by the state of the honey (crystallized or not) and the glucose content, which means the aw was higher in the crystallized honey than in either uncrystallized or liquid honey. In liquid honey, glucose is more bound to water than in crystallized honey, thus contributing to the increase in aw [53]. Our results are consistent with those already reported by Gleiter et al. [54], suggesting that our honey samples are possibly safe for fermentation, as an aw of less than 0.60 is considered insufficient for osmophilic yeasts growth [55].

### 3.3. Phytochemical Constituents and Antioxidant Activities

Owing to their ability to chelate metal ions (Cu^+^, Fe^3+^), donate hydrogen (H-atom transfer) and electrons (one-electron transfer), and thus form stable radical intermediates, flavonoids and many other phenolic compounds are recognized as potent antioxidant molecules with a stronger efficacy to fight overproduced reactive oxygen species and other free radicals [56]. Natural antioxidant components, mainly polyphenols present in plant extracts, bee products, and other functional foods, protect against oxidative stress and associated health disorders. For these reasons, the assessment of polyphenol and flavonoid content has become the trend of green chemistry and attracts the attention of many researchers and industries worldwide. In this sense, the present results showed that the amount of phenolic content varied significantly from 20.92 to 155.89 mg GAE/100 g for H13 and H7, respectively, which was in the range of values reported by El Menyiy et al. [57] for different monofloral Moroccan honeys, ranging from 17.35 mg GAE/100 g in *Peganum harmala* honey samples to 219.02 mg GAE/100 g in *Acacia tortilis* honey samples. For flavonoid content, H9 (5.52 mg QE/100 g) and H23 (20.69 mg QE/100 g) presented, respectively, the lowest and highest flavonoid contents. High amount of phenolics and flavonoids occur in dark amber samples; by contrast, their low content occurs in white honey samples. Besides the plant species, the variations showncould be related to the plant origin and pedoclimatic characteristics of each harvested station [46].

Regarding the antioxidant activity, three different and complementary assays (TAA, DPPH, and ABTS tests) were used to evaluate distinct antioxidant mechanisms. It is recommended to use more than one method for the general assessment of the antioxidant capacity of functional food extracts [58,59]. Additionally, the used reagents and the specific experimental conditions react differently with regard to the specific individual antioxidant component contained in the examined extracts. Therefore, the wide differences in the chemical structures of phenolic and nonphenolic antioxidant molecules and their concentrations, as well as the complex chemistry of the involved test, lead to the differences in the antioxidant assay results [60]. As is clearly presented in Table 4, evaluated samples (H1–H24) exhibited different antioxidant activities regardless of the methodused.

In addition to phenolic compounds, organic honeys contain many other antioxidant molecules, including vitamin C. Owing to its ability to donate electrons, this compound has been documented as a powerful antioxidant molecule with multiple human benefits [61]. Vitamin C concentration in honey is affected by different factors, including the floral origin, the pollen density, honey enzymes, and the preservation condition [27]. Our honey samples contain concentrations of ascorbic acid ranging between 8.01 ± 0.12 and 23.26 ± 0.74 mg/100 g. The lowest value corresponds to sample H16, while the highest value corresponds to sample H13. These values are similar to those reported for Moroccan monofloral honeys with a predominance of *Bupleurum spinosium* pollen [46] and for Pakistanian *Ziziphus lotus* honeys [62], but are lower than those of monofloral honeys from the south-western part of Saudi Arabia [63].

Concerning the total antioxidant activity, the *Ziziphus lotus* honey sample (H13),whichhad the lowest phenolic content (20.92 mg GAF/100 g) and the poorest antiradical-scavenging ability (IC_50_ = 45.20 mg/mL), expressed the lowest total antioxidant capacity 34.18 mg AAE/g, followed by extra-white (H14), white (H8), and extra-white (H9) honeys. It is most important to mention that even containing a low amount of TPC and a poorest DPPH radical inhibition, H13 exhibited the second-highest ABTS free-radical-scavenging capacity (IC_50_ =8.19 mg/mL). Although these results seem contradictory, the obtained data could be due to several variables, including the individual antioxidant molecules present in the honey solution and their specific ability to inhibit DPPH and ABTS free radicals [64]. Substantially, different DPPH and ABTS inhibition activities were displayed by several phenolic compounds, viz *p*-coumaric acid and ferulic acid were inactive against DPPH, while displaying an IC_50_ equal to 23.46 and 21.62 ± 0.10 μg/mL for ABTS, respectively. According to the same study, the authors showed that galangine was more effective against ABTS free radical (IC_50_ = 8.73 ± 0.02 μg/mL) than DPPH radical (IC_50_= 17.02 ± 0.02 μg/mL) and caffeic acid (IC_50_ = 4.73 ± 0.02 μg/mL for ABTS *vs* IC_50_= 4.91 ± 0.00 μg/mL for DPPH) [65,66], which prove and reaffirm the impact of individual molecules on the antioxidant capacity of functional foods, including organic honey, and their specific interactions with different free radicals.

Generally, the antioxidant capacities of natural products/extracts are strongly dependent on the chemical structure of their phenolic components. For example, supposing identical patterns of methoxy and hydroxyl substitutions, hydroxycinnamic acids are more effective against ABTS radical cations than hydroxybenzoic acids [67]. Thus, the structural characteristics of bioactive ingredients, as well as the possible interactions between them, play a crucial role in the mechanism of antioxidant action, which makes the subject of the antioxidant activity too complex to be explained just in terms of the quantity of phenolic and flavonoid contents. Moreover, other nonphenolic compounds, mainly terpenoids and ascorbic acid, may critically inhibit the ABTS radical cation (ABTS^•+^) and, thus, support the obtained results [68,69]. By contrast, dark amber honeys, H4, H17, and H7, displayed the best total antioxidant activity, with values of 131.20, 124.82, and 115.80 mg AAE/g, respectively. In addition to polyphenols, the total antioxidant capacities of different honeys may be influenced by their complex compositions on other bioactive compounds, such as polysaccharides, amino acids, active peptides, vitamins, and other antioxidant microelements [70].

DPPH has commonly been used as a reactive hydrogen acceptor for the assessment of the radical scavenging activity of various antioxidant molecules from medicinal herbs, functional food extracts, and/or synthetic active compounds [71]. As indicated in Table 4, the concentration of honey required to inhibit 50% of DPPH^•^ radical showed a significant variation between the samples. *Ziziphus lotus* honey (H7) from Ain Cheggag had the highest amount of polyphenols (155.89 ± 0.03 mg GAE/100 g) and exhibited the best antiradical activity (IC_50_ = 8.14 mg/mL). However, *Ziziphus lotus* honey (H13), harvested from Guigou, which had the lowest amount of polyphenols and presented the lowest DPPH-scavenging capacity, IC_50_ = 45.20 mg/mL. Despite these samples being grouped as monofloral *Ziziphus lotus* honeys, they showed different aspects concerning their antiradical activity, which reaffirms the impact of the secondary nectar flora origin and pedoclimatic characteristics of each harvested station. The discrepancies noted may also have been due to the composition of each tested honey on specific individual phenolic and nonphenolic antioxidant components [64]. These results were in agreement with our previous data, in which eightmonofloral honey samples (*Bupleurum spinosum*)collected from different regions of Morocco showed wide variations in phenol content, total flavonoids, ascorbic acid, TAC, and antiradical activity [46]. The obtained values are higher (lower antiradical activities) than thoseobserved for Trolox 10.81 ± 0.1 µg/mL and were in line with those reported previously for 14 Moroccan monofloral honeys, wherein the IC_50_ values ranged between 4.79 and 45.64 mg/mL in the *Acacia tortilis* and *Citrus sinensis* honey samples, respectively [57]. Substantially potent DPPH scavenging capacities were displayed by various phenolic acids viz ascorbic acid (99.1%), gallic acid (92.0%), caffeic acid (91.2%), and syringic acid (90.4%) [72]. These molecules have been identified previously in Moroccan honeys [46,73], which supports the hypothesis of their contribution to the antiradical activity of the examined samples.

Regarding the ABTS scavenging capacities, polyfloral honey of the Bekrit area (H23),whichhad the highest phenolic and flavonoid content (106.71 mg GAE/100 g and 20.69 mg QE/100 g, respectively), exhibited the best ABTS radical-scavenging activity (IC_50_ = 3.34 mg/mL), while the sample harvested from the Oulad Youssef area (H8) presented the lowest inhibition percentage of the ABTS free radical (IC_50_ = 32.76 mg/mL). In addition to the possible influence of predominant and secondary nectar composition, the amount of wide variation observed in the examined samples reaffirm the impact of geographical origins on the phytochemical composition of honey samples and, thus, their antioxidant potential. The reported activities are lower than that documented by Trolox (IC_50_ = 23.15 µg/mL) and are within the range of values reported for 17 mono- and polyfloral honeys collected from different ecogeographical regions of Morocco, with IC_50_ values between 4.49 to 31.00 mg/mL for Oregano and jujube honeys, respectively [15]. The demonstrated variation in the antioxidant activity of the analyzed honeys was in line with the data reported for 19 honey samples from Poland and 15 monofloral honey samples harvested from different ecogeographic regions of Brazil [74,75].

The relationship between total phenolic content, total flavonoid content, and the antioxidant activities of the examined honey samples (TAA, DPPH, and ABTS scavenging activities) was performed using the Pearson correlation coefficient (Table 5). A significant (r = 0.902, *p* < 0.001) positive correlation was obtained between the total phenolic content and total antioxidant activity. Moreover, a negative correlation was observed between total phenolic content and IC_50_ values of DPPH (r = −0.810, *p* < 0.001) and ABTS (r = −0.240), indicating the intervention of the mentioned biocompounds in the antioxidant activity process of organic honey. Nevertheless, as expected, this correlation was not significant for ABTS. On the other hand, a nonsignificant positive correlation has been established between the IC_50_ values of ABTS and DPPH (r = 0.096). Similar data were documented elsewhere [76].

Examined honey samples displayed powerful and wide differences in antioxidant activities, which are probably attributed to their distinct phytochemical composition, mainly, their antioxidant components. These results allow us to highlight the beneficial use of organic honey as a bioactive functional food in the daily human diet.

### 3.4. Sugars Content

In this study, 13 sugars were identified and quantified by GC-FID after their oximation and derivatizations. A representative chromatogram is presented in the Supplementary File.

Based on Table 6, the identified sugars consisted of two monosaccharides, six disaccharides, and five trisaccharides. Table 6 also shows the means, standard deviations, and ranges of each individual sugar in all of the analyzed honey samples. Our study confirms that glucose and fructose werethe major quantified sugars in all samples. Fructose wasthe predominant sugar in honey samples, with an average value of 40.16 g/100 g, followed by glucose, maltose, turanose, melibiose + isomaltose, raffinose, and sucrose, which werealso detected in all investigated samples. However, panose, melezitose, maltotriose, erlose, palatinose, and trehalose are quantified as minor sugars and were not detected in all of the analyzed honeys. The sugar composition depends highly on the types of flowers used by the bees, as well as the ecogeographical characteristics of the growing area [75].

The total sugar content of the honey samples varied between 67.06 and 79.85%. The polyfloral honey (H20) from the Skoura zone contained the highest level of total sugars.However, the lowest content of total sugars was observed in H7, named asfour, from Ain cheggag. The result of the total sugar content (79.85%) of the average organic atlas honeys agrees with those from Algeria, as reported by Ouchmoukh et al. [77].

More specifically, fructose was the most abundant sugar and varied between 35.99 and 42.57%, followed by glucose (with a range of 33.77–40.16%) (Table 6). The monosaccharide sugar content (glucose and fructose), with values between 61.3% and 73.02%, are within the limits authorized by the Council of the European Union (2002) (>60%) [43]—except in the case of the H15 sample of the ElhajOutat area, which presented a value of 58.29 g/100 g. The results showed that the fructose–glucose ratio also varied considerably between samples from 1.10 to 1.51 for sample H2 and sample H14, respectively.Such sugar contents are associated with the botanical source, geographical origin, processing and storage conditions, and also the climate, among many other factors.

Crystallization of honey is also slower when thefructose/glucose ratio is high. In our study, the Rhamnaceaehoney (H15: jujube honey) had a low amount of glucose (24.52%) and the proportion of fructose represented 33.77%. This honey type is always in a liquid physical state because the fructose/glucose ratio exceeds 1.35% [78]. In addition to its impact on the sensory characteristics and the physical state of honey, the fructose/glucose ratio remains an essential criterion that conditions its use in certain critical physiological cases, such as lipid and glucose metabolic dysfunctions. Pasupuleti and coworkers documented that the fructose contained in honey improved hyperglycemia in experimental diabetic animals and diabetic patients [6]. Likewise, dietary fructose has been found to improve glycemic status by enhancing glucokinase activity and thereby catalyzing the conversion of glucose to glucose-6-phosphate and increasing hepatic glucose uptake (storage of glucose as glycogen by the liver tissue) [79]. For that, besides phenolic compounds and many other macro/microantioxidant nutrients, fructose-rich honey might be at least useful to enhance human physiological abilities and prevent several metabolic disorders, including diabetes.

Other honey types (buplevre, asfour, jujube, and thymus) showed the same average amount of fructose. The concentrations of fructose in Middle Atlas honeys are quite the same compared to Algerian honeys [36]. However, eucalyptus and citrus honeys were reported as richer sources of monosaccharides [80,81]. The composition of monosaccharides from the same flower source may vary due to seasonal climate variability, as well as different geographic origins.

About the disaccharides, maltose, turanose, sucrose, melibiose, palatinose, and trehalosewerethe major ones detected. Of these, the first four disaccharides werepresent in all of the studied honey samples, and maltose represents the main disaccharide. The average obtained for our samples was3.78%. In Brazilian honeys, the average amount was reported at 3.05% [82], whereas in Spanish honeys, the published mean was3.96% [77].

Sucrose waspresent in 23 samples, ranging between 0.03 and 0.91%.For other Moroccan honey samples, it was found that this disaccharide was only detected in heather honey (mean value 4.12%) [45].

The sucrose content values of our investigated samples werein the range of those obtained for Turkish honey samples [83], and werelower than those obtained by El Sohaimy and coworkers (3.31 ± 0.23, 3.43 ± 0.12, and 3.59 ± 0.20 g/100 g, respectively, for Yemanian, Egyptian, and Saudanian honey) [84]. It was reported that sucrose content can decrease during the storage of honey due to the presence of the invertase [81]. According to Leite et al. [82], the reason for the variable levels of sucrose could be due to a transglycosylation reaction initiated by the transfer of the a-D-glucopyranosyl unit from sucrose to an acceptor molecule. The sucrose contents of the tested honeysamplesdid notexceed the highest limit recommended by the European Community directive (5%) [43]. Overall, our results confirm that the studied honeys wereat an advanced stage of maturation and wereauthentic because sucrose is the most important sugar from a legislative point of view.

Additionally, the trehalose content wasdetected in H4, H7, H11, H18, H19, and H20, with a very low quantity and the mean value didnot exceed 0.01%. However, this sugar has been found at a high level (mean value of 0.09%) in the Moroccan eucalyptus honey analyzed by Terrab et al. [45]. Melibiose wasdetected in all samples analyzed, with 0.04% and 1.08% in the present study, whereas values of melibiose for Brazilian honeys were reported to range between 0.05 and 0.15% [82].

Trisaccharides (erlose, raffinose, melezitose, maltotriose, and panose) are nonreducing saccharides (except panose). Among the trisaccharides, erlosewaspresent in 22 honey samples.The same was true for raffinose, maltotriose, panose and melezitose, which were generally present in all of the honey samples. The values for erlosewerein the range of 0.09–2.19% and were higher than those reported by Pérez-Arquillué et al. [85]. This sugar waspresent in 22 honey samples and was not detected in one monofloral honey, named lharra (H16). The monofloral honey H15 had the highest value. Raffinose, present in all samples, didnot exceed 0.32%. Melezitose, present in 19 samples, ranged between 0.02% and 0.46%. This sugar is usually indicative of honeydew honey. Sanz et al. [86] obtained a high concentration of melezitose (6.57%) in 10 Spanish honeydew samples. The percentage of melezitosewaslow, indicating that these are nectar honeys.

### 3.5. Principal Component Analysis (PCA)

**Figure 1:**
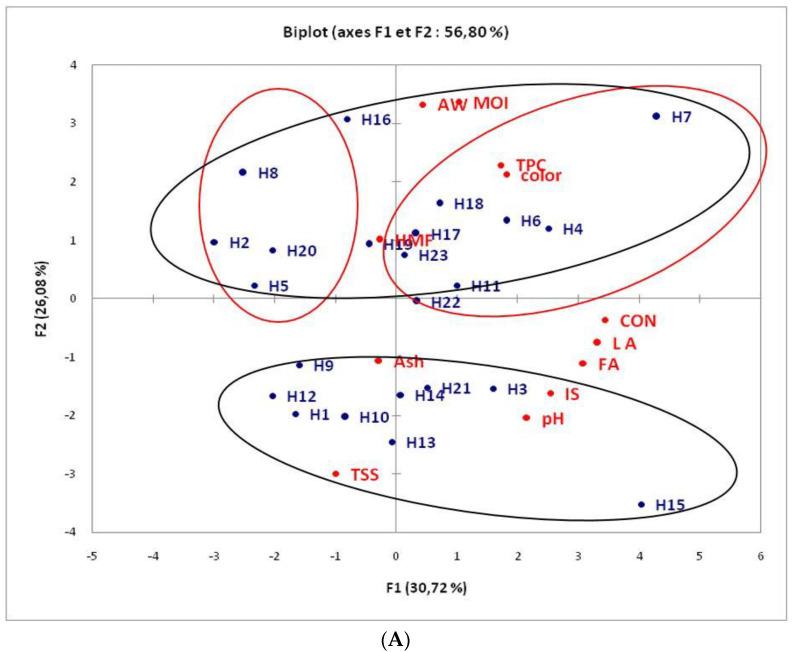

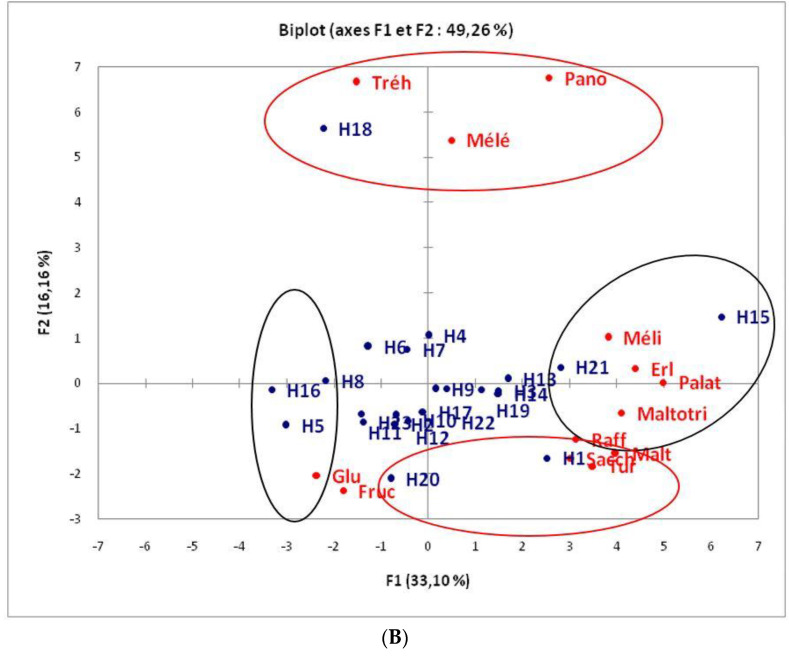
Principal component analysis (PCA) of honey:(**A**) PC1:30.72%, PC2:26.08.07%; and (**B**) PC1:33.10%, PC2:16.16%. Relationship between honey physicochemical and sugar profile (score and loading biplot). Aw: water activity, TPC: total phenolic compounds, color, pH, HMF: hydroxy methyl furfural, TSS: total soluble solids, MOI: moisture, LA: lactonic acidity, FA: free acidity, CON: conductivity, ash, IS: saccharase index, tréh: trehalose, Pano: panose, Mélé: melezitose, Méli: melibiose, Erl: erlose, Palat: palatinose, Fruc: fructose, Glu: glucose, Raff: raffinose, Malt: maltose, Maltotri: maltotriose, Tur: turanose, Sacc:saccharose.

In this study, PCA was used to determine the physicochemical and sugar profiles of honey. Two experiments (Figure 1A,B) were conducted: In the first one (Figure 1A), the PCA was conductedfrom the physicochemical data; in the second experiment (Figure 1B), the PCA was conductedfrom the sugar profile data. The first axis (F1) of PCA explained the largest part of the variance (30.72%). As shown in Figure 1A, the honeys wereseparated along this axis (F1) into two groups, (H2, H5, H8, and H20) and (H4, H6, H7, H17, H18, and H23), according to their amounts of phenolic compounds, color, water activity, moisture, and HMF attributes. (H4, H6, H7, H17, H18, and H23 werericher in polyphenols, and HMF and had high levels of color). The second component (F2, 26.08%, Figure 1A) distinguished two groups: (H1, H3, H9, H10, H12, H13, and H14) and the rest according to the ash content, total soluble solids, pH, saccharase index, and free acidity. Indeed, the (H1, H3, H9, H10, H12 H13, H14, and H15) group wasassociated with large amounts of acid compounds, total soluble solids, and with lesser amounts of phenolic compounds, HMF, and the pigment responsible for the color of honey, explaining 56.80% of the total variance. pH had a positive correlation with conductivity (0.522 **), free acidity (0.481 **), andlactonic acidity (0.620 *), and a negative correlation with water activity (−0.238 *). HMF had a positive correlation with color (0.430 **) and saccharase index (0.531 **). As shown in Figure 1B, the honeys wereseparated along this axis (F1, 33.10) into two groups, (H16 and H5) and (H21 and H15), according to their sugarcontents. H21 and H15 werericher in palatinose, erlose, melezitose, and maltotriose. This other group, (H5 and H16), corresponding to the elharra honey collected from the Oumjniba area, wasless rich in palatinose, erlose, melezitose, and maltotriose. The second component (F2, 16,1%, Figure 1B) distinguished two groups, (H1 and H20) and H18, according to the quantitative sugar characteristics. Indeed, H18 wasassociated with a high level of trehalose, panose, and meletriose sugar and with lesser amounts of raffinose and sucrose. Unexpectedly, the other group (H1 and H20) wasassociated with a higher content of raffinose and sucrose and a lesser level, or near-absence, of trehalose, panose, and maltotriose. There wasa correlation between fructose and panose (−0.433 *).Melibiose had a positive correlation with maltose (0.471 *), palatinose (0.5112 *), and raffinose (0.468 *). Our study showed a positive correlation between turanose and maltose (0.593 **) and turanose and raffinose (0.674 **). It also showed a positive correlation between palatinose and saccharose (0.569 *) and palatinose and maltotriose (0.586 **).Finally, there were very significant correlations between melezitose and trehalose(0.460 **) and trehalose and panose (0.596 **). It is most important to notethat all of the grouped honeys did not share the same characteristic regarding the harvest period, which excludes the hypothesis of its impact on the proximate and phytochemical compositions of the investigated organic honeys.

## 4. Conclusions

The physicochemical status of the 23 analyzed honey samples meets international standards and proved good beekeeping practices and correct honey conditioning and storage. Furthermore, the obtained results showed that the antioxidant capacities of the evaluated organic honeys vary with plant sources. Further investigations must be conducted to develop rules of preservation treatments necessary to increase the shelf-life of honey. In addition, our results recall other studies and more complete approaches to the nutritional and physicochemical profiles and health-benefit values of honeys produced in different Moroccan regions in order to create specific chemical-labeling methods and to adopt minimum and obligatory Moroccan norms that should be required for producing honey with good quality and to combat potential possible adulterations.

## Figures and Tables

**Table 1:** Honey samples, origins, and harvesting period.

Code	Local Denomination	Geographical Origin	Latitude	Longitude	Altitude (m)	Harvest Period
H1	Thyme	Serghina	33°20′19″ N	4°29′26″ W	1578	June 2018
H2	Rosemary	ImouzzerMarmoucha	33°24′44″ N	4°17′45″ W	1447	April 2018
H3	Jujube	Ain Cheggag	33°48′27″ N	5°06′59″ W	749	August 2018
H4	Buplevre	El Mers	33°26′10″ N	4°27′10″ W	1550	August 2018
H5	ElHarra	Oumjniba, Boulemane	33°20′09″ N	4°40′50″ W	1950	May 2018
H6	Buplevre, Chouk	Oumjniba, Boulemane	33°20′38″ N	4°36′21″ W	1784	August 2018
H7	Asfour	Ain Cheggag	33°51′13″ N	5°07′24″ W	697	August 2018
H8	Buplevre	Oulad Ali Youssef, Imouzzer	33°27′43″ N	3°58′28″ W	1362	September 2018
H9	Thyme	El Mers	33°26′36″ N	4°26′34″ W	1487	June 2018
H10	El Harmel	Enjil	33°12′42″ N	4°36′56″ W	1683	July 2018
H11	Asfour	Ahl Sidi Lahcen, Sefrou	33°46′55″ N	4°40′48″ W	960	August 2018
H12	Multiflower	Boulemane Centre	33°21′31″ N	4°43′42″ W	1730	June 2018
H13	Fijel	Guigou	33°28′47″ N	4°51′46″ W	1578	July 2018
H14	Jujube	Outat El Haj	33°20′44″ N	3°45′26″ W	841	August 2018
H15	Jujube	Outat El Haj	33°22′44″ N	3°45′03″ W	834	July 2018
H16	Lharra	Oumjniba, Boulemane	33°19′38″ N	4°40′33″ W	1912	May 2018
H17	Buplevre	Bouyblan	33°39′06″ N	4°03′57″ W	1948	September 2018
H18	Jujube	Missour	33°02′32″ N	3°58′20″ W	886	July 2018
H19	Buplevre	ImouzzerMarmoucha	33°25′22″ N	4°18′57″ W	1412	September 2018
H20	El Harra	Skoura	33°29′23″ N	4°36′03″ W	1070	May2018
H21	Jujube	Ifrane	33°35′23″ N	5°09′33″ W	1426	July 2018
H22	Jujube	Oued Ifrane	33°18′05″ N	5°29′38″ W	904	July 2018
H23	Thyme	Bekrit	33°03′10″ N	5°13′03″ W	1868	May 2018

**Table 2:** Frequency of distribution of taxa in the 23 analyzed honeys.

		Predominant Pollen (PP)		Secondary Pollen (SP)	
Pollen Taxa	Honey Group	Honey Number	Frequency PP (%)	Honey Number	Frequency SP (%)
*Ziziphus lotus*	G1	6	51–93	-	-
Rhamnaceae	G2	2	43–68	1	11
*Sinapis arvensis*	G3	2	54–63	1	20
Fabaceae	G4	1	73	4	10–37
*Rosmarinus officinalis*	G5	1	64	-	-
Lamiaceae	G6	1	64	4	11–34
*Thymus vulgaris*	G7	1	55	-	-
*Ammi visnaga*	G8	1	53	-	-
Apiaceae	G9	1	41	3	11–12
Rosaceae		-	-	9	11–36
*Olea europea*		-	-	3	10–18
*Rubus*		-	-	3	17–28
Cistaceae		-	-	2	10–14
Brassicaceae		-	-	2	13–15
Rutaceae		-	-	2	14–21
Oleaceae		-	-	1	35
Anacardiaceae	G10	-	-	1	10
*Plantago ovata*		-	-	1	36
*Salix*		-	-	1	18
*Diplotaxisharra*		-	-	1	28
Chenopodiaceae		-	-	1	20
*Echium vulgare*		-	-	1	17

**Table 3:** Physicochemical analysis of honey samples.

Honey Samples	Moisture (%)	Free Acidity (mEq/kg)	LactonicAcidity (mEq/kg)	Total Acidity (mEq/kg)	pH	Ash Content (%)	Electrical Conductivity (µs/cm)	HMF	TSS (%)	Saccharase Index	Honey Color	PfundScale (mm)	Activity of Water
H1	14.2 ± 0.4 ^a^	28.65 ± 1.3 ^ef^	9.84 ± 0.5 ^c^	38.49 ± 1.8 ^i^	4.16 ± 0.18 ^a^	0.24 ± 0.1 ^i^	110 ± 0.02 ^a^	5.1 ± 2.3 ^ab^	85.8 ± 0.3 ^h^	26.7 ± 1.7 ^hij^	White	22.3 ± 0.5 ^e^	0.563 ± 0.01 ^abc^
H2	16.1 ± 0.4 ^cde^	17.42 ± 1.3 ^c^	7.07 ± 0.5 ^a^	24.49 ± 1.8 ^k^	3.93 ± 0.18 ^a^	0.28 ± 0.1 ^def^	100 ± 0.02 ^a^	5.5 ± 2.3 ^ab^	83.9 ± 0.3 ^e^	3.5 ± 1.7 ^a^	White	29.5 ± 0.5 ^h^	0.522 ± 0.01 ^abc^
H3	14.8 ± 0.4 ^ab^	36.31 ± 1.3 ^gh^	18.29 ± 0.5 ^i^	54.6 ± 1.8 ^c^	4.37 ± 0.18 ^a^	2.44 ± 0.1 ^ef^	620 ± 0.02 ^l^	3.5 ± 2.3 ^ab^	85.2 ± 0.3 ^gh^	23.8 ± 1.7 ^efghi^	Amber	92.9 ± 0.5 ^o^	0.560 ± 0.01 ^a^
H4	17.8 ± 0.4 ^g^	35.76 ± 1.3 ^g^	16.43 ± 0.5 ^h^	52.19 ± 1.8 ^d^	4.31 ± 0.18 ^a^	0.162 ± 0.1 ^j^	460 ± 0.02 ^h^	1.2 ± 2.3 ^ab^	82.2 ± 0.3 ^bc^	36.3 ± 1.7 ^k^	Dark Amber	125.7 ± 0.5 ^s^	0.551 ± 0.01 ^abc^
H5	16.2 ± 0.4 ^cde^	15.12 ± 1.3 ^b^	8.59 ± 0.5 ^b^	23.71 ± 1.8 ^kl^	3.94 ± 0.18 ^a^	0.007 ± 0.1 ^abcdef^	160 ± 0.02 ^b^	2.7 ± 2.3 ^ab^	83.8 ± 0.3 ^e^	17.9 ± 1.7 ^cd^	White	26.9 ± 0.5 ^g^	0.560 ± 0.01 ^abc^
H6	17.4 ± 0.4 ^fg^	34.48 ± 1.3 ^g^	16.16 ± 0.5 ^h^	50.64 ± 1.8 ^e^	4.28 ± 0.18 ^a^	0.21 ± 0.1 ^a^	410 ± 0.02 ^g^	3 ± 2.3 ^ab^	82.6 ± 0.3 ^cd^	29.3 ± 1.7 ^ij^	Dark Amber	118.1 ± 0.5 ^q^	0.591 ± 0.01 ^abc^
H7	19.2 ± 0.4 ^h^	38.88 ± 1.3 ^h^	22.84 ± 0.5 ^j^	61.72 ± 1.8 ^b^	4.24 ± 0.18 ^a^	0.43 ± 0.1 ^cdef^	820 ± 0.02 ^n^	4.9 ± 2.3 ^ab^	80.8 ± 0.3 ^a^	28.8 ± 1.7 ^ij^	Dark Amber	123.8 ± 0.5 ^r^	0.549 ± 0.01 ^bc^
H8	19.0 ± 0.4 ^h^	12.34 ± 1.3 ^a^	10.51 ± 0.5 ^cd^	22.85 ± 1.8 ^m^	3.89 ± 0.18 ^a^	1.48 ± 0.1 ^g^	100 ± 0.02 ^a^	1.2 ± 2.3 ^ab^	81 ± 0.3 ^a^	4.3 ± 1.7 ^a^	White	25.7 ± 0.5 ^f^	0.534 ± 0.01 ^abc^
H9	16.0 ± 0.4 ^cde^	29.98 ± 1.3 ^ef^	11.16 ± 0.5 ^d^	41.14 ± 1.8 ^h^	4.04 ± 0.18 ^a^	3.88 ± 0.1 ^h^	160 ± 0.02 ^b^	2 ± 2.3 ^ab^	84 ± 0.3 ^e^	18.3± 1.7 ^cde^	Extra white	16.4 ± 0.5 ^d^	0.533 ± 0.01 ^ab^
H10	15.2 ± 0.4 ^bc^	21.07 ± 1.3 ^d^	14.73 ± 0.5 ^g^	35.8 ± 1.8 ^j^	4.37 ± 0.18 ^a^	0.28 ± 0.1 ^k^	320 ± 0.02 ^e^	N.D	84.8 ± 0.3 ^fg^	25.5 ± 1.7 ^ghij^	White	27.6 ± 0.5 ^g^	0.546 ± 0.01 ^ab^
H11	16.5 ± 0.4 ^de^	42.57 ± 1.3 ^i^	12.39 ± 0.5 ^e^	54.96 ± 1.8 ^c^	4.12 ± 0.18 ^a^	0.06 ± 0.1 ^ef^	490 ± 0.02 ^ij^	7.6 ± 2.3 ^ab^	83.5 ± 0.3 ^e^	20.1 ± 1.7 ^cg^	Amber	89.4 ± 0.5 ^m^	0.529 ± 0.01 ^abc^
H12	14.2 ± 0.4 ^a^	28.19 ± 1.3 ^ef^	8.62 ± 0.5 ^b^	36.81 ± 1.8 ^j^	3.97 ± 0.18 ^a^	0.16 ± 0.1 ^ab^	160 ± 0.02 ^b^	5.2 ± 2.3 ^ab^	85.8 ± 0.3 ^h^	18.7 ± 1.7 ^cde^	White	33.2 ± 0.5 ^i^	0.507 ± 0.01 ^a^
H13	15.7 ± 0.4 ^bcd^	36.73 ± 1.3 ^gh^	14.19 ± 0.5 ^g^	50.92 ± 1.8 ^e^	4.27 ± 0.18 ^a^	0.26 ± 0.1 ^abcde^	490 ± 0.02 ^ij^	4.2 ± 2.3 ^ab^	84.3 ± 0.3 ^ef^	23.9 ± 1.7 ^efghi^	Water white	8.1 ± 0.5 ^b^	0.542 ± 0.01 ^a^
H14	15.7 ± 0.4 ^bcd^	35.45 ± 1.3 ^g^	14.44 ± 0.5 ^g^	49.89 ± 1.8 ^e^	4.27 ± 0.18 ^a^	0.18 ± 0.1 ^ef^	500 ± 0.02 ^jk^	4.4 ± 2.3 ^ab^	84.3 ± 0.3 ^ef^	24.9 ± 1.7 ^fghi^	Extra white	11.00 ± 0.5 ^c^	0.536 ± 0.01 ^abc^
H15	16.0 ± 0.4 ^cde^	49.02 ± 1.3 ^j^	28.92 ± 0.5 ^k^	77.94 ± 1.8 ^a^	6.34 ± 0.18 ^c^	0.11 ± 0.1 ^bcde^	680 ± 0.02 ^m^	N.D	84 ± 0.3 ^e^	30.5 ± 1.7 ^j^	White	22.3 ± 0.5 ^e^	0.563 ± 0.01 ^abc^
H16	19.4 ± 0.4 ^h^	31.31 ± 1.3 ^f^	9.57 ± 0.5 ^bc^	40.88 ± 1.8 ^h^	3.98 ± 0.18 ^a^	0.32 ± 0.1 ^abcd^	220 ± 0.02 ^d^	4.6 ± 2.3 ^ab^	80.6 ± 0.3 ^a^	10.4 ± 1.7 ^b^	White	29.5 ± 0.5 ^h^	0.522 ± 0.01 ^abc^
H17	16.1 ± 0.4 ^cde^	27.52 ± 1.3 ^e^	14.29 ± 0.5 ^g^	41.81 ± 1.8 ^h^	4.35 ± 0.18 ^a^	0.07 ± 0.1 ^f^	480 ± 0.02 ^i^	32.8 ± 2.3 ^d^	83.9 ± 0.3 ^e^	8 ± 1.7 ^ab^	Amber	92.9 ± 0.5 ^o^	0.560 ± 0.01 ^a^
H18	18.0 ± 0.4 ^g^	29.16 ± 1.3 ^ef^	13.66 ± 0.5 ^fg^	42.82 ± 1.8 ^g^	4.31 ± 0.18 ^a^	0.24 ± 0.1 ^ab^	410 ± 0.02 ^g^	4.5 ± 2.3 ^ab^	82 ± 0.3 ^b^	20.6 ± 1.7 ^cdefg^	Dark amber	125.7 ± 0.5 ^s^	0.551 ± 0.01 ^abc^
H19	16.1 ± 0.4 ^cde^	28.94 ± 1.3 ^ef^	14.05 ± 0.5 ^fg^	42.99 ± 1.8 ^g^	4.32 ± 0.18 ^a^	0.10 ± 0.1 ^cdef^	350 ± 0.02 ^f^	35.7 ± 2.3 ^d^	83.9 ± 0.3 ^e^	5.7 ± 1.7 ^a^	White	26.9 ± 0.5 ^g^	0.560 ± 0.01 ^abc^
H20	16.1 ± 0.4 ^cde^	21.87 ± 1.3 ^d^	10.28 ± 0.5 ^cd^	32.15 ± 1.8 ^j^	4.08 ± 0.18 ^a^	0.24 ± 0.1 ^abc^	200 ± 0.02 ^c^	10.9 ± 2.3 ^c^	83.9 ± 0.3 ^e^	5.7 ± 1.7 ^a^	Dark amber	118.1 ± 0.5 ^q^	0.591 ± 0.01 ^abc^
H21	16.4 ± 0.4 ^bcd^	28.11 ± 1.3 ^ef^	10.47 ± 0.5 ^cd^	38.58 ± 1.8^i^	4.34 ± 0.18 ^b^	0.18 ± 0.1 ^cdef^	510 ± 0.02 ^ij^	5 ± 2.3 ^ab^	83.6 ± 0.3 ^e^	22.3 ± 1.7 ^dh^	Dark amber	123.8 ± 0.5 ^r^	0.549 ± 0.01 ^bc^
H22	17.0 ± 0.4 ^cde^	30.92 ± 1.3 ^ef^	12.26 ± 0.5 ^e^	43.18 ± 1.8 ^f^	4.14 ± 0.18 ^a^	0.2 ± 0.1 ^ab^	310 ± 0.02 ^k^	4.4 ± 2.3 ^ab^	83 ± 0.3 ^d^	19.4 ± 1.7 ^cf^	White	25.7 ± 0.5 ^f^	0.534 ± 0.01 ^abc^
H23	14.2 ± 0.4 ^ef^	30.79 ± 1.3 ^ef^	13.09 ± 0.5 ^f^	43.88 ± 1.8 ^f^	4.16 ± 0.18 ^a^	0.28 ± 0.1 ^bcde^	110 ± 0.02 ^e^	5.1 ± 2.3 ^ab^	85.8 ± 0.3 ^h^	16 ± 1.7 ^c^	Extra white	16.4 ± 0.5 ^d^	0.533 ± 0.01 ^ab^
Min	14.2 ± 0.4	12.34 ± 1.3 ^a^	7.07 ± 0.5 ^a^	22.85 ± 1.8	3.89 ± 0.18 ^a^	0.007 ± 0.1	100 ± 0.02	ND	80.6 ± 0.3	3.5 ± 1.7	Water white	8.1 ± 0.5	0.507 ± 0.01
Max	19.4 ± 0.4	49.02 ± 1.3 ^j^	28.92 ± 0.5 ^k^	77.94 ± 1.8	6.34 ± 0.18	3.88 ± 0.1	820 ± 0.02	35.7 ± 2.3	85.8 ± 0.3	36.3 ± 1.7	Dark Amber	125.7 ± 0.5	0.591 ± 0.01

All values are expressed as means of triplicate determinations ± standard deviation(SD). TSS = total soluble solid. Values in the same column followed by the same letter are not significantly different by Tukey’s multiple-range test (*p* < 0.05).

**Table 4:** Phytochemical constituents and antioxidant activities of honey samples.

Samples	Phenolics (mg GAE/100 g)	Flavonoids (mg QE/100 g)	Ascorbic Acid (mg/100 g)	TAA (mg AAE/g)	DPPH (IC_50_ = mg/mL)	ABTS (IC_50_ = mg/mL)
H1	67.96 ± 0.03 ^h^	9.72 ± 0.03^bc^	10.33 ± 0.16 ^bc^	54.89 ± 0.15 ^c^	17.32 ± 0.88 ^d^	21.52 ± 0.21 ^c^
H2	72.19 ± 0.03 ^j^	15.40 ± 0.03 ^e^	10.48 ± 0.03 ^bc^	66.20 ± 0.61 ^ab^	17.51 ± 0.75 ^d^	24.74 ± 0.08 ^ab^
H3	110.70 ± 0.03 ^q^	7.17 ± 0.03 ^b^	9.98 ± 0.72 ^b^	118.94 ± 0.18 ^i^	13.54 ± 0.32 ^bc^	19.06 ±1.74 ^c^
H4	152.95 ± 0.03 ^t^	8.85 ± 0.03 ^b^	15.02 ± 0.3 ^g^	131.20 ± 0.85 ^j^	12.47 ± 0.21 ^b^	16.74 ± 0.12 ^c^
H5	60.01 ± 0.03 ^f^	16.37 ± 0.03 ^ef^	12.77 ± 0.79 ^e^	69.22 ± 0.26 ^ab^	17.06 ± 0.89 ^d^	14.67 ± 0.02 ^b^
H6	124.84 ± 0.03 ^s^	11.35 ± 0.03 ^bc^	15.73 ± 0.18 ^g^	82.27 ± 2.37 ^d^	9.34 ± 0.26 ^a^	13.65 ± 0.15 ^b^
H7	155.89 ± 0.03 ^u^	10.98 ± 0.03 ^bc^	16.5 ± 0.44 ^h^	115.80 ± 3.66 ^i^	8.14 ± 0.33 ^a^	18.82 ± 0.07 ^c^
H8	52.92 ± 0.03 ^d^	15.51 ± 0.03 ^e^	18.73 ± 0.17 ^i^	41.89 ± 0.04 ^b^	19.68 ± 0.45 ^d^	32.76 ± 0.43 ^d^
H9	38.98 ± 0.03 ^c^	5.52 ± 0.03 ^a^	9.92 ± 0.56 ^b^	45.05 ± 0.23 ^b^	23.89 ± 0.57 ^d^	18.63 ± 1.46 ^c^
H10	70.23 ± 0.03 ^i^	17.20 ± 0.03 ^ef^	20.59 ± 0.08 ^j^	81.94 ± 0.26 ^d^	15.05 ± 0.20 ^bc^	22.65 ± 0.73 ^ab^
H11	93.75 ± 0.03 ^o^	12.09 ± 0.03 ^d^	13.75 ± 0.2 ^f^	96.87 ± 0.37 ^e^	14.51 ± 0.18 ^bc^	28.90 ± 1.04 ^d^
H12	62.15 ± 0.03 ^g^	13.81 ± 0.03 ^d^	11.88 ± 0.24 ^d^	52.05 ± 0.04 ^c^	18.99 ± 0.04 ^d^	17.62 ± 0.07 ^c^
H13	20.92 ± 0.03 ^a^	13.73 ± 0.03 ^d^	23.26 ± 0.35 ^k^	34.18 ± 0.15 ^a^	45.20 ± 0.65 ^f^	8.19 ± 0.11 ^b^
H14	21.54 ± 0.03 a	5.97 ± 0.0 ^a^	19.85 ± 0.45 ^j^	40.77 ± 0.26 ^b^	39.48 ± 0.43 ^e^	30.24 ± 1.97 ^d^
H15	24.34 ± 0.03 ^b^	8.57 ± 0.03 ^b^	11.09 ± 0.11 ^cd^	49.82 ± 0.61 ^c^	36.76 ± 0.81 ^e^	27.41 ± 1.16 ^d^
H16	56.95 ± 0.03 ^e^	17.85 ± 0.03 ^ef^	8.01 ± 0.5 ^a^	50.41 ± 0.01 ^c^	20.92 ± 0.05 ^d^	18.64 ± 0.31 ^c^
H17	123.54 ± 0.03 ^r^	16.31 ± 0.03 ^ef^	16.86 ± 0.58 ^h^	124.82 ± 7.48 ^i^	9.02 ± 0.17 ^a^	21.45 ± 0.25 ^ab^
H18	90.56 ± 0.03 ^n^	13.82 ± 0.03 ^d^	14.15 ± 0.86 ^f^	95.27 ± 3.87 ^e^	12.56 ± 0.63 ^b^	10.93 ± 0.07 ^b^
H19	78.68 ± 0.03 ^k^	12.98 ± 0.03 ^d^	14.92 ± 0.9 ^g^	62.01 ± 0.04 ^ab^	16.09 ± 0.61 ^bc^	21.03 ± 0.11 ^ab^
H20	80.58 ± 0.03 ^l^	6.26 ± 0.03 ^a^	11.54 ± 0.71 ^d^	65.76 ± 0.25 ^ab^	10.11 ± 0.34 ^a^	28.73 ± 0.07 ^d^
H21	81.70 ± 0.03 ^l^	15.02 ± 0.03 ^e^	15.41 ± 0.1 ^g^	69.07 ± 0.05 ^ab^	11.59 ± 0.15 ^b^	18.74 ± 0.16 ^c^
H22	83.15 ± 0.03 ^m^	17.02 ± 0.03 ^ef^	18.04 ± 0.23 ^i^	70.08 ± 0.01 ^ab^	11.33 ± 0.03 ^a^	28.47 ± 1.06 ^d^
H23	106.71 ± 0.03 ^p^	20.69 ± 0.03 ^i^	17.3 ± 0.3 ^h^	105.56 ± 7.35 ^f^	9.81 ± 0.92 ^a^	3.34 ± 0.07 ^a^
Min	20.92 ± 0.03	5.52 ± 0.03	8.01 ± 0.5	34.18 ± 0.15	8.14 ± 0.33	8.19 ± 0.11
Max	155.89 ± 0.03	20.69 ± 0.03	23.26 ± 0.35	131.20 ± 0.85	45.20 ± 0.65	32.76 ± 0.43
Trolox (µg/mL)	-	-		-	10.81 ± 0.1 ^b^	23.15 ± 4.0 ^ab^

Values in the same column followed by the same letter are not significantly different by Tukey’s multiple-range test (*p* < 0.05).

**Table 5:** Pearson coefficients between phenolics, TAA, DPPH, and ABTS.

	Phenolics	TAA	DPPH	ABTS
Phenolics	1	0.902 ***	−0.810 ***	−0.240
TAA	0.902 ***	1	−0.681 ***	−0.260
DPPH	−0.810 ***	−0.681 ***	1	0.096
ABTS	−0.240	−0.260	0.096	1

*** Correlation is significant at the level *p* < 0.001.

**Table 6:** Sugars values (g/100 g) of honey types.

Samples	Fructose	Glucose	Maltose+	Turanose+	Melibiose and Isomaltose	Sucrose	Trehalose	Palatinose	Raffinose	Erlose	Melezitose	Maltotriose	Panose
H1	37.22 ± 3.2 ^a^	30.47 ± 2.14 ^abcd^	5.17 ± 1.32 ^a^	1.96 ± 0.64 ^a^	0.41 ± 0.38 ^a^	0.34 ± 0.1 ^c^	ND	0.16 ± 0.1 ^b^	0.32 ± 0.12 ^a^	0.81 ± 0.16 ^e^	0.03 ± 0.01 ^a^	0.07 ± 0.02 ^abcd^	0.08 ± 0.01 ^abcdef^
H2	36.1 ± 3.32 ^a^	32.79 ± 2.14 ^d^	3.12 ± 1.32 ^a^	0.99 ± 0.64 ^a^	0.21 ± 0.1 ^a^	0.33 ± 0.1 ^c^	ND	ND	0.21 ± 0.12 ^a^	1.24 ± 0.16 ^fg^	0.03 ± 0.01 ^a^	0.12 ± 0.02 ^cde^	0.04 ± 0.01 ^abc^
H3	36.36 ± 3.32 ^a^	30.57 ± 2.14 ^abcd^	3.63 ± 1.32 ^a^	1.53 ± 0.64 ^a^	0.73 ± 0.38 ^a^	0.07 ± 0.01 ^a^	ND	0.09 ± 0.02 ^ab^	0.3 ± 0.12 ^a^	0.66 ± 0.16 ^de^	0.04 ± 0.01 ^a^	0.1 ± 0.02 ^bcd^	0.14 ± 0.010 ^ef^
H4	38.25 ± 3.32 ^a^	27.99 ± 2.14 ^abcd^	4.04 ± 1.32 ^a^	1.42 ± 0.64 ^a^	0.56 ± 0.38 ^a^	0.03 ± 0.0 ^a^	0.03 ± 0.01 ^a^	ND	0.29 ± 0.12 ^a^	0.11 ± 0.02 ^ab^	0.46 ± 0.01 ^d^	0.04 ± 0.02 ^abc^	0.07 ± 0.01 ^abcdef^
H5	38.7 ± 3.32 ^a^	31.79 ± 2.14 ^cd^	3.25 ± 1.32 ^a^	0.73 ± 0.64 ^a^	0.25 ± 0.1 ^a^	0.05 ± 0.01 ^a^	ND	ND	0.16 ± 0.12 ^a^	0.25 ± 0.16 ^abc^	ND	ND	0.03 ± 0.01 ^ab^
H6	36 ± 3.32 ^a^	26.73 ± 2.14 ^abcd^	3.16 ± 1.32 ^a^	1.25 ± 0.64 ^a^	0.06 ± 0.01 ^a^	0.03 ± 0.01 ^a^	ND	0.05 ± 0.02 ^ab^	0.25 ± 0.12 ^a^	0.11 ± 0.02 ^ab^	0.33 ± 0.01 ^c^	ND	0.07 ± 0.01 ^abcde^
H7	34.93 ± 3.32 ^a^	26.37 ± 2.14 ^abcd^	3.77 ± 1.32 ^a^	0.88 ± 0.64 ^a^	0.33 ± 0.1 ^a^	0.06 ± 0.01 ^a^	0.03 ± 0.01 ^a^	ND	0.27 ± 0.12 ^a^	0.14 ± 0.02 ^ab^	0.06 ± 0.01 ^a^	0.06 ± 0.02 ^abcd^	0.12 ± 0.01 ^cdef^
H8	37.32 ± 3.32 ^a^	28.53 ± 2.14 ^ad^	2.68 ± 1.32 ^a^	0.72 ± 0.64 ^a^	0.2 ± 0.1 ^a^	0.08 ± 0.01 ^a^	ND	ND	0.23 ± 0.12 ^a^	0.46 ± 0.16 ^abcd^	0.02 ± 0.01 ^a^	0.04 ± 0.02 ^abc^	0.05 ± 0.01 ^abcd^
H9	36.29 ± 3.32 ^a^	31.49 ± 2.14 ^bcd^	4.32 ± 1.32 ^a^	0.91 ± 0.64 ^a^	0.37 ± 0.1 ^a^	0.1 ± 0.01 ^ab^	ND	0.15 ± 0.02 ^b^	0.23 ± 0.12 ^a^	0.61 ± 0.16 ^cde^	0.02 ± 0.01 ^a^	0.07 ± 0.02 ^abcd^	0.13 ± 0.01 ^def^
H10	37.51 ± 3.32 ^a^	31.76 ± 2.14 ^cd^	4.95 ± 1.32 ^a^	0.95 ± 0.64 ^a^	0.48 ± 0.38 ^a^	0.07 ± 0.01 ^a^	ND	0.14 ± 0.02 ^ab^	0.22 ± 0.12 ^a^	0.26 ± 0.16 ^abc^	ND	ND	0.08 ± 0.01 ^abcdef^
H11	36.81 ± 3.32 ^a^	26.74 ± 2.14 ^abcd^	3.34 ± 1.32 ^a^	0.81 ± 0.64 ^a^	0.2 ± 0.1 ^a^	0.06 ± 0.01 ^ab^	0.01 ± 0.01 ^a^	ND	0.28 ± 0.12 ^a^	0.25 ± 0.16 ^abc^	ND	0.02 ± 0.02 ^ab^	0.04 ± 0.01 ^abc^
H12	37.98 ± 3.32 ^a^	31.6 ± 2.14 ^bcd^	3.73 ± 1.32 ^a^	0.93 ± 0.64 ^a^	0.27 ± 0.1 ^a^	0.26 ± 0.1 ^bc^	ND	0.11 ± 0.02 ^ab^	0.24 ± 0.12 ^a^	0.49 ± 0.16 ^bcde^	0.06 ± 0.01 ^a^	0.05 ± 0.02 ^abcd^	0.08 ± 0.01 ^abcde^
H13	37.86 ± 3.32 ^a^	25.27 ± 2.14 ^ab^	3.82 ± 1.32 ^a^	1.07 ± 0.64 ^a^	0.42 ± 0.38 ^a^	0.19 ± 0.1 ^abc^	ND	0.16 ± 0.02 ^b^	0.27 ± 0.12 ^a^	1.36 ± 0.16 ^fg^	0.09 ± 0.01 ^ab^	0.13 ± 0.02 ^de^	0.12 ± 0.01 ^cdef^
H14	39.22 ± 3.32 ^a^	25.93 ± 2.14 ^abc^	4.2 ± 1.32 ^a^	1.07 ± 0.64 ^a^	0.42 ± 0.38 ^a^	0.2 ± 0.1 ^abc^	ND	0.16 ± 0.02 ^b^	0.25 ± 0.12 ^a^	1.4 ± 0.16 ^g^	0.09 ± 0.01 ^ab^	0.12 ± 0.02 ^cde^	0.11 ± 0.01 ^bcdef^
H15	33.77 ± 3.32 ^a^	24.52 ± 2.14 ^a^	4.54 ± 1.32 ^a^	1.15 ± 0.64 ^a^	1.08 ± 0.38 ^a^	0.74 ± 0.1 ^c^	ND	0.44 ± 0.02 ^c^	0.28 ± 0.12 ^a^	2.19 ± 0.16 ^h^	0.11 ± 0.01 ^ab^	0.13 ± 0.02 ^de^	0.23 ± 0.01 ^g^
H16	35.75 ± 3.32 ^a^	32.26 ± 2.14 ^cd^	1.95 ± 1.32 ^a^	0.57 ± 0.2 ^a^	0.2 ± 0.1 ^a^	0.03 ± 0.01 ^a^	ND	ND	0.22 ± 0.12 ^a^	ND	ND	ND	0.06 ± 0.01 ^abcd^
H17	37.03 ± 3.32 ^a^	28.11 ± 2.14 ^abcd^	4.37 ± 1.32 ^a^	1.09 ± 0.64 ^a^	0.44 ± 0.38 ^a^	0.04 ± 0.01 ^a^	ND	ND	0.27 ± 0.12 ^a^	0.09 ± 0.02 ^a^	0.02 ± 0.01 ^a^	0.09 ± 0.02 ^abcd^	0.08 ± 0.01 ^abcde^
H18	37.49 ± 3.32 ^a^	30.41 ± 2.14 ^abcd^	3.09 ± 1.32 ^a^	0.73 ± 0.64 ^a^	0.28 ± 0.1 ^a^	0.08 ± 0.01 ^a^	0.05 ± 0.01 ^a^	ND	0.18 ± 0.12 ^a^	0.4 ± 0.02 ^abcd^	0.33 ± 0.01 ^c^	ND	0.3 ± 0.01 ^h^
H19	39.16 ± 3.32 ^a^	30.79 ± 2.14 ^abcd^	4.1 ± 1.32 ^a^	1.11 ± 0.64 ^a^	0.5 ± 0.38 ^a^	0.19 ± 0.1 ^abc^	0.02 ± 0.01 ^a^	0.16 ± 0.02 ^b^	0.27 ± 0.12 ^a^	0.28 ± 0.16 ^abc^	0.19 ± 0.01 ^b^	0.21 ± 0.02 ^f^	0.1 ± 0.01 ^bcdef^
H20	40.16 ± 3.32 ^a^	32.86 ± 2.14 ^d^	3.47 ± 1.32 ^a^	1.22 ± 0.64 ^a^	0.22 ± 0.1 ^a^	0.91 ± 0.1 ^f^	0.03 ± 0.01 ^a^	0.07 ± 0.02 ^ab^	0.23 ± 0.12 ^a^	0.65 ± 0.16 ^de^	ND	0.03 ± 0.02 ^ab^	ND
H21	35.63 ± 3.32 ^a^	29.61 ± 2.14 ^abcd^	4.33 ± 1.32 ^a^	1.23 ± 0.64 ^a^	0.04 ± 0.01 ^a^	0.49 ± 0.1 ^d^	ND	0.29 ± 0.02 ^b^	0.22 ± 0.12 ^a^	2.03 ± 0.16 ^h^	0.17 ± 0.01 ^ab^	0.18 ± 0.02 ^cd^	0.16 ± 0.01 ^ef^
H22	38.18 ± 3.32 ^a^	26.06 ± 2.14 ^abc^	3.95 ± 1.32 ^a^	1.02 ± 0.64 ^a^	0.39 ± 0.1 ^a^	0.13 ± 0.01 ^ab^	ND	0.17 ± 0.02 ^b^	0.19 ± 0.12 ^a^	1.09 ± 0.16 ^f^	0.09 ± 0.01 ^ab^	0.08 ± 0.02 ^abcd^	0.08 ± 0.01 ^abcd^
H23	38.78 ± 3.32 ^a^	27.58 ± 2.14 ^abcd^	3.96 ± 1.32 ^a^	0.85 ± 0.64 ^a^	0.29 ± 0.1 ^a^	0.07 ± 0.01 ^a^	ND	0.09 ± 0.02 ^ab^	0.19 ± 0.12 ^a^	0.39 ± 0.16 ^abcd^	0.02 ± 0.01 ^a^	0.03 ± 0.02 ^ab^	0.03 ± 0.01 ^ab^
Min	33.77 ± 3.32	24.52 ± 2.14	2.68 ± 1.32	0.57 ± 0.2	0.04 ± 0.01	0.03 ± 0.01	ND	ND	0.16 ± 0.12	ND	ND	ND	ND
Max	40.16 ± 3.32	32.86 ± 2.14	5.17 ± 1.32	1.96 ± 0.64	1.08 ± 0.38	0.91 ± 0.1	0.05 ± 0.01	0.44 ± 0.02	0.32 ± 0.12	2.03 ± 0.16	0.46 ± 0.01	0.21 ± 0.02	0.3 ± 0.01

Values in the same column followed by the same letter are not significantly different by Tukey’s multiple-range test (*p* < 0.05).

## Data Availability

The data presented in this study are available on request from the corresponding author.

## Supplementary Materials

The following supporting information can be downloaded at: https://www.mdpi.com/article/10.3390/foods11213362/s1, Supplementary File: Chromatogram sugars profile of honey samples
