# Supplementary material for: Organic Honey from the Middle Atlas of Morocco: Physicochemical Parameters, Antioxidant Properties, Pollen Spectra, and Sugar Profiles
Source: Foods. 2022 Oct 26;11(21):3362. doi: 10.3390/foods11213362 (PMC9658496; doi:10.3390/foods11213362)
Supplement: Supplementary file 1 [file foods-11-03362-s001.zip › foods-1948801-supplementary.pdf]

Chromatogram

Page 1 of 1

Sample Name : thym sem4119 Sample #: 010
FileName : C:\GC\Data\ROUTINE\semaine4119\24670 sem4119.raw
Date : 12/18/2019 12:18:38 PM
Method : Time of Injection: 10/8/2019 1:36:10 AM
Start Time : 0.00 min End Time : 54.19 min Low Point : 0.00 mV High Point : 400.00 mV
Plot Offset: 0.00 mV Plot Scale: 400.0 mV

Chromatogram:Sugars profil for thym honey

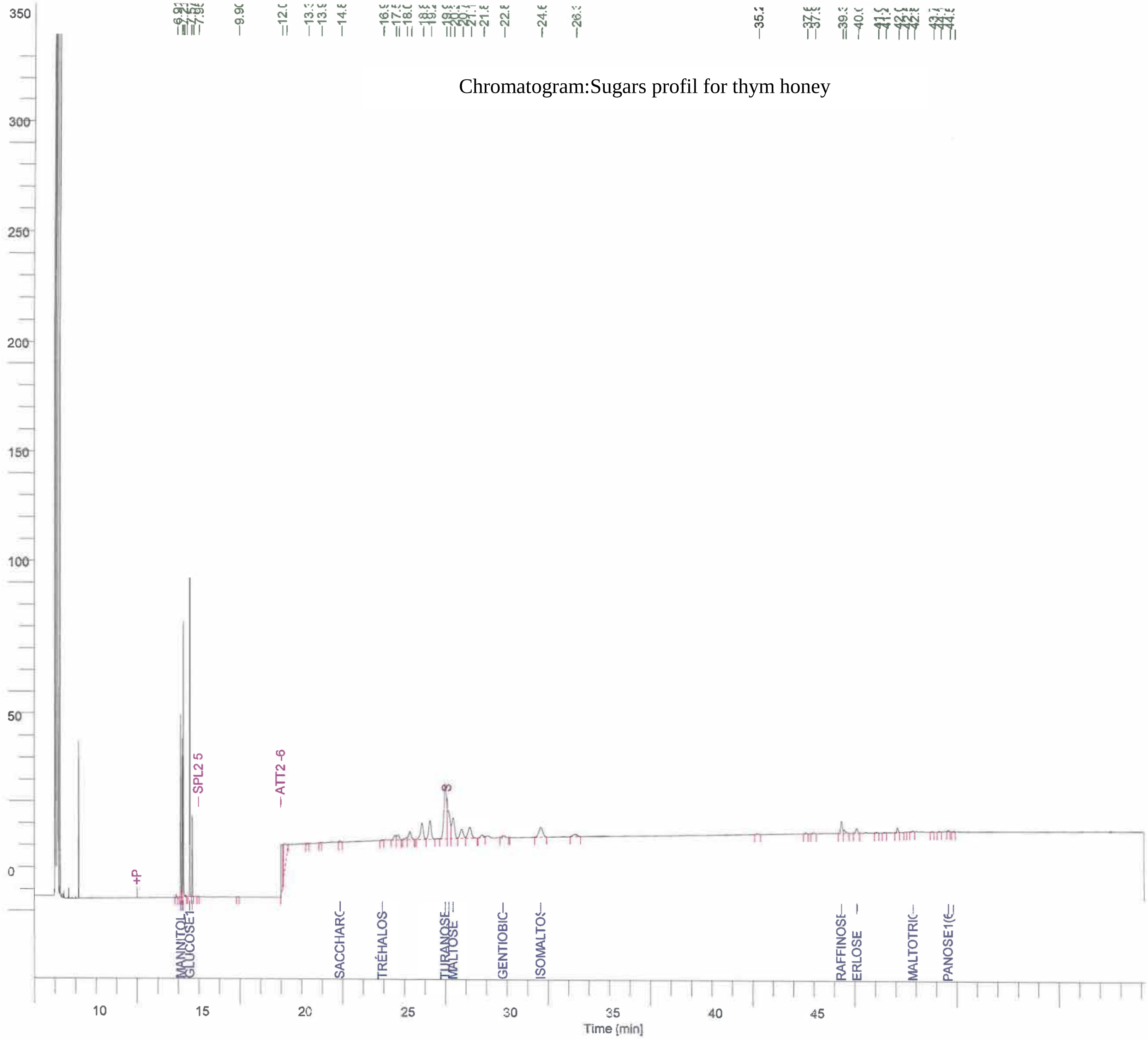

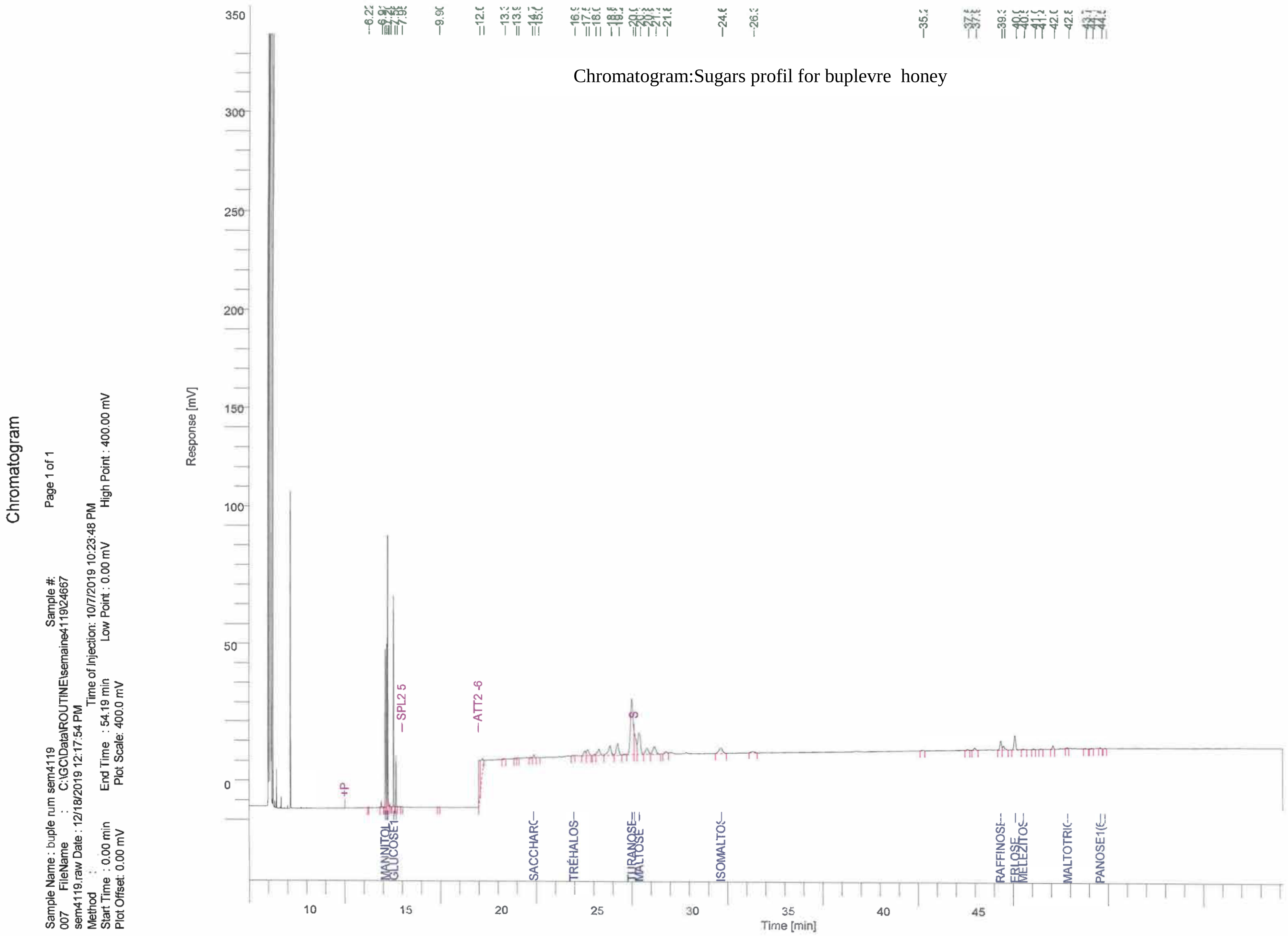

Chromatogram:Sugars profil for buplevre honey

Chromatogram: Sugars profil for jujube honey

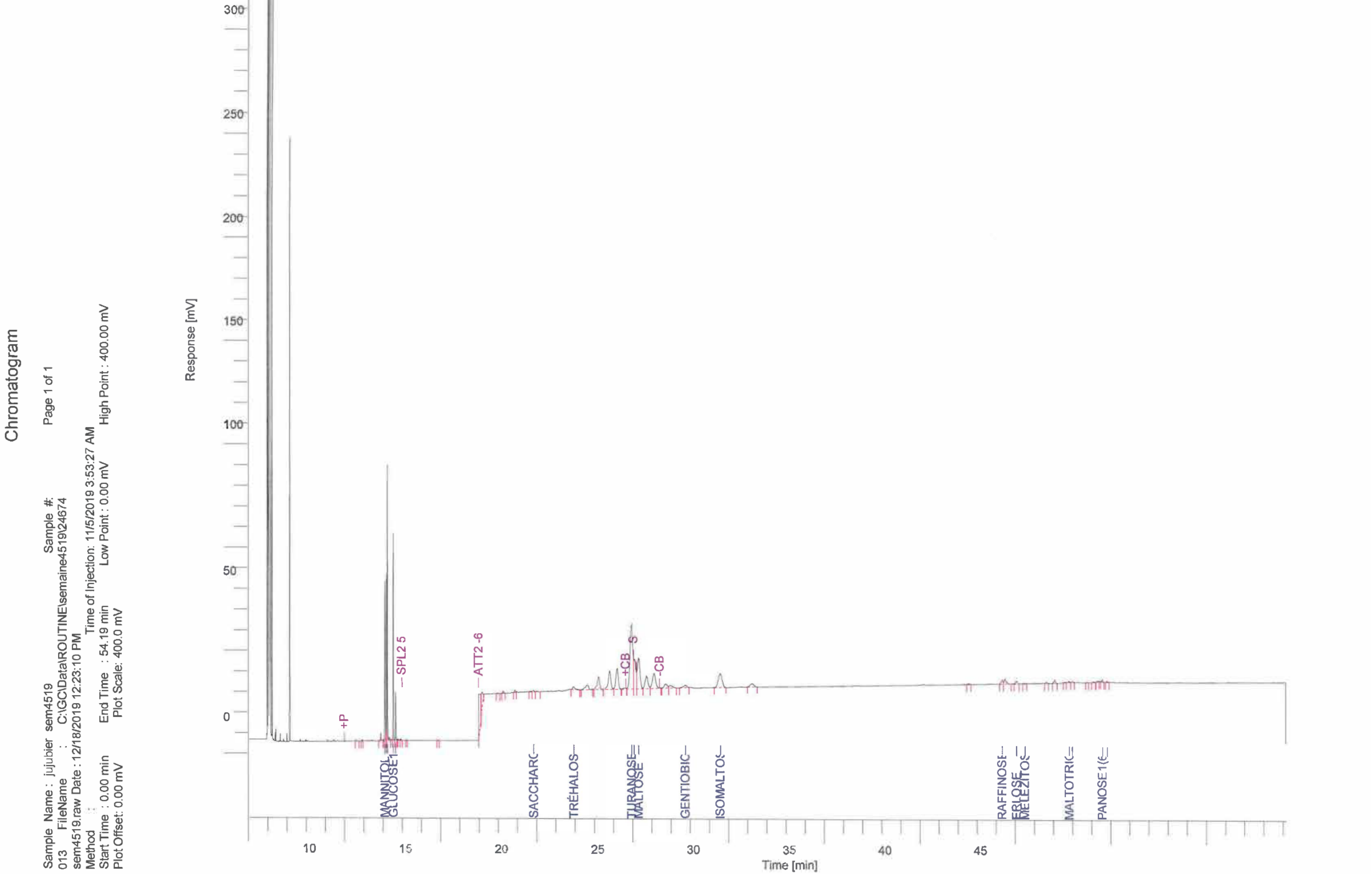

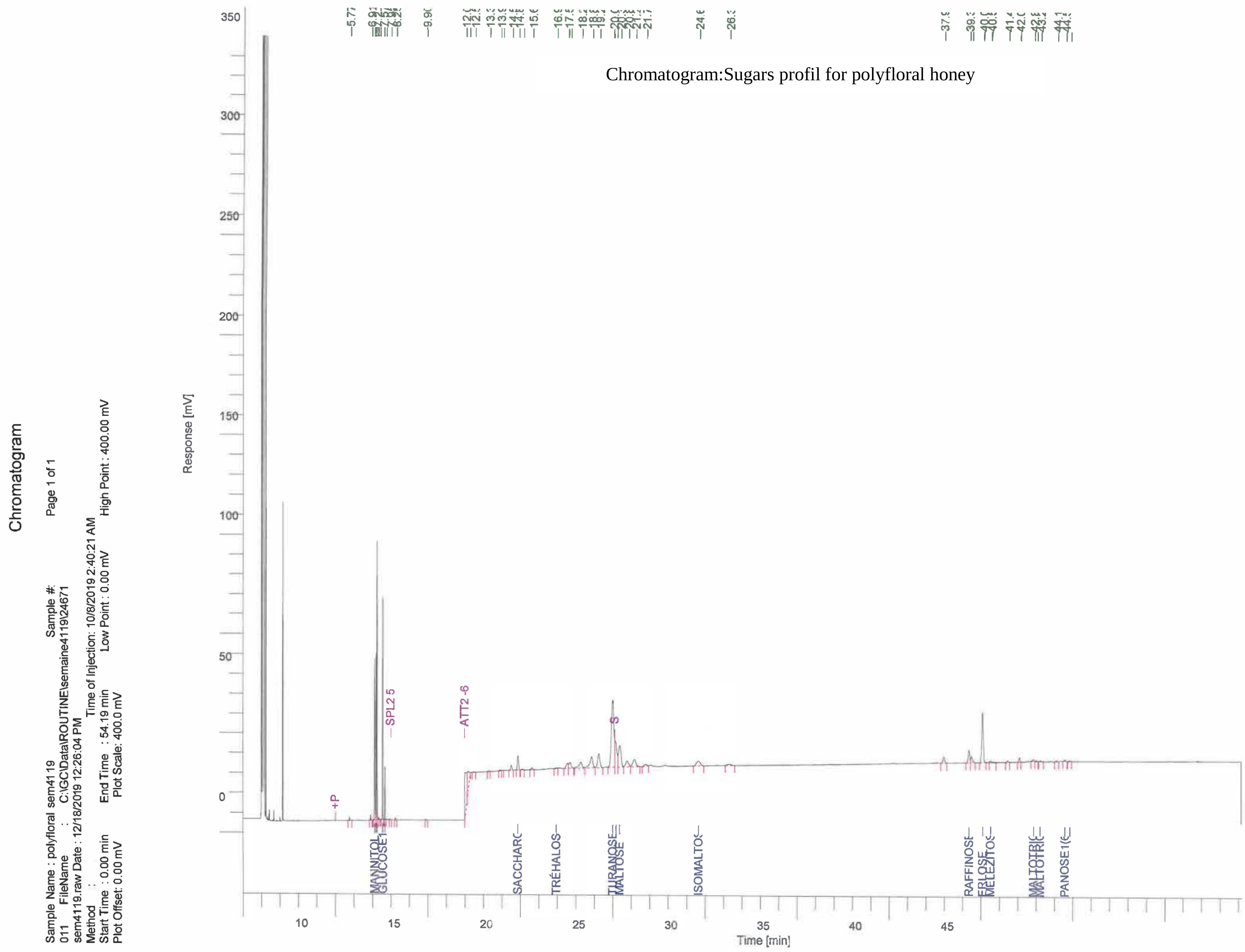

Chromatogram:Sugars profil for polyfloral honey
